# Simple methodology to visualize whole-brain microvasculature in three dimensions

**DOI:** 10.1117/1.NPh.8.2.025004

**Published:** 2021-04-19

**Authors:** Katiana Khouri, Danny F. Xie, Christian Crouzet, Adrian W. Bahani, David H. Cribbs, Mark J. Fisher, Bernard Choi

**Affiliations:** a University of California, Irvine, Beckman Laser Institute and Medical Clinic, Irvine, California, United States; b University of California, Irvine, Graduate Program in Mathematical, Computational, and Systems Biology, Irvine, California, United States; c University of California, Irvine, Department of Biomedical Engineering, Irvine, California, United States; d University of California, Irvine, Institute for Memory Impairments and Neurological Disorders, Irvine, California, United States; e University of California, Irvine, Department of Neurology, Orange, California, United States; f University of California, Irvine, Department of Pathology and Laboratory Medicine, Irvine, California, United States; g University of California, Irvine, Department of Anatomy and Neurobiology, Irvine, California, United States; h University of California, Irvine, Edwards Lifesciences Center for Advanced Cardiovascular Technology, Irvine, California, United States; i University of California, Irvine, Department of Surgery, Irvine, California, United States

**Keywords:** microvasculature, optical clearing, light sheet, whole-brain, cerebrovascular

## Abstract

**Significance:** To explore brain architecture and pathology, a consistent and reliable methodology to visualize the three-dimensional cerebral microvasculature is beneficial. Perfusion-based vascular labeling is quick and easily deliverable. However, the quality of vascular labeling can vary with perfusion-based labels due to aggregate formation, leakage, rapid photobleaching, and incomplete perfusion.

**Aim:** We describe a simple, two-day protocol combining perfusion-based labeling with a two-day clearing step that facilitates whole-brain, three-dimensional microvascular imaging and characterization.

**Approach:** The combination of retro-orbital injection of Lectin-Dylight-649 to label the vasculature, the clearing process of a modified iDISCO+ protocol, and light-sheet imaging collectively enables a comprehensive view of the cerebrovasculature.

**Results:** We observed ∼threefold increase in contrast-to-background ratio of Lectin-Dylight-649 vascular labeling over endogenous green fluorescent protein fluorescence from a transgenic mouse model. With light-sheet microscopy, we demonstrate sharp visualization of cerebral microvasculature throughout the intact mouse brain.

**Conclusions:** Our tissue preparation protocol requires fairly routine processing steps and is compatible with multiple types of optical microscopy.

## 1 Introduction

Neurons rely on the complex vascular network to supply them with metabolites and oxygen. Dysfunction of the microvasculature is associated with several cerebral disorders, including schizophrenia, traumatic brain injury, dementia, and stroke.^1^^–^^3^ Fundamental to exploring these relationships is a consistent and reliable methodology to visualize the three-dimensional cerebral microvasculature under normal and pathological conditions. Researchers would benefit in particular from whole-brain models of the cerebrovascular structure. The crucial and challenging task of creating useful whole-organ visualizations requires the pairing of compatible labeling, clearing, and imaging techniques using repeatable and robust methodologies and equipment.

Vascular labeling techniques include secondary antibody staining via diffusion, transgenic endothelial markers, and vessel labeling perfusion-based methods. While secondary antibody staining requires long labeling periods (up to seven days for mouse hemibrains),^4^^,^^5^ perfusion-based vascular labeling is quick and easily deliverable.^6^^–^^8^ However, the quality of vascular labeling can vary with perfusion-based labels due to aggregate formation, leakage, rapid photobleaching, and incomplete perfusion.^6^^,^^9^^–^^11^ For example, for immunolabeling, antibody diffusion from the exposed surfaces inwards can lead to heterogeneous vessel labeling, especially for deeper vessel structures.^12^ For cardiac perfusion methods, capillary labeling can be heterogeneous, presumably due to insufficient pressure to maintain consistent perfusion of all capillaries.^13^

Tissue clearing procedures have progressed rapidly, enabling whole-organ clearing using aqueous solvents, organic solvents, or hydrogel infusion with electrophoretic tissue clearing.^4^^,^^14^^,^^15^ Recent advances in light-sheet microscopy (LSM) enable detailed three-dimensional imaging of intact cleared organs with excellent optical sectioning capabilities.^4^^,^^12^^,^^16^ LSM can achieve short whole-organ imaging time (∼1 to 2 h for a mouse brain) while maintaining μm-level resolution and mitigating photobleaching. Recent reports demonstrate whole-organ vascular visualization in the brain and teeth.^14^^,^^17^ However, the use of complicated clearing procedures necessitating lengthy labeling techniques and non-standard equipment has precluded the routine use of these methodologies for scientific research.^6^^,^^18^ Here, we describe a simple protocol combining perfusion-based labeling with a two-day clearing step that facilitates whole-brain, three-dimensional microvascular imaging and characterization.

## 2 Materials and Methods

### 2.1 Animal Models

All animal experiments were performed under a protocol approved by the Institutional Animal Care and Use Committee at the University of California, Irvine. Whole-brain vessel labeling experiments were performed using an adult transgenic mouse model (Tie2-GFP) expressing green fluorescent protein (GFP) in vascular endothelial cells [Tg (TIE2-GFP)287Sato/J Stock No. 003658 Jackson Laboratory, Bar Harbor, Maine] (n=4, age range: 5 to 21 months). Whole-brain vessel labeling was also performed on a separate cohort consisting of three-month-old C57BL/6J mice (n=3).

### 2.2 Exogenous Fluorescent Dyes

Vessel labeling agents can be distinguished by their selective cell binding sites and solubility. *Lycopersicon esculentum* Lectin (Lectin), a glycoprotein with a binding affinity to glycoprotein moieties found in the vascular endothelium, has several fluorescent derivatives. In this study, we performed vessel labeling with Lectin-Dylight-649 (Vector Laboratories DL-1178, Burlingame, California).

### 2.3 Vessel Labeling

For all mice, anesthesia was performed using 5% isoflurane inhalation followed by IP administration of a ketamine-xylazine cocktail (90  mg/kg ketamine, 10  mg/kg xylazine). A diluted aliquot (50  μl dye, 150  μl saline) of Lectin Dylight-649 was administered via retro-orbital injection. The retro-orbital route was selected over tail-vein injection due to the relative ease of exogenous agent administration^19^ and to our prior experience with this method for photosensitizer administration.^20^^,^^21^ The dye was allowed to circulate for 30 min, followed by an IP injection of euthasol (195 mg sodium pentobarbital). Cardiac perfusion of 10 ml saline followed by 10 ml of 10% formalin was immediately performed to flush out the blood and fix the tissue, respectively.

### 2.4 Brain Extraction

After vessel labeling, the brains were harvested and immersed in 10% formalin for 24 h in the dark, then transferred to a 0.1% sodium azide solution (balance PBS) and stored at 4°C.

### 2.5 Tissue Clearing

We modified the published iDISCO+ protocol^4^ to achieve brain clearing in 4 h (brain slices) or in two days (whole brains) with one round of dehydration and elimination of the secondary antibody labeling procedures.

#### 2.5.1 Brain slices

Whole brains (n=4) were cut into 1-mm-thick coronal brain slices. One coronal slice from each brain was selected for this study. The slices were dehydrated with a cascading methanol treatment (20%, 40%, 60%, 80%, 100%, 100%, balance sterilized water) for 20 min each at room temperature. During this treatment, samples were placed in black 5-ml Eppendorf tubes to avoid potential photobleaching effects of ambient light. They were then submerged in 66% dichloromethane (DCM) (Sigma 270997-12X100ML) (balance methanol) for 1 h and left on the nutator to mix. Two 15-min washes of 100% DCM were done to remove excess methanol. The samples were submerged in dibenzyl ether (DBE) (Sigma 108014-1KG) and kept in the dark until imaging.

#### 2.5.2 Whole brains

A similar protocol described for the brain slices was used for clearing of the whole brains (n=3). Primary differences include (1) application of the cascading methanol treatment (20%, 40%, 60%, 80%, 100%, 100%, balance sterilized water) for 1 h, and (2) submerging of the intact brains in 66% DCM (Sigma 270997-12X100ML) (balance methanol) for 3 h.

### 2.6 Imaging

#### 2.6.1 Comparison of Tie2-GFP endogenous and Lectin-Dylight-649 exogenous fluorescence

Spectral images were acquired with the Leica True Confocal Scanner SP8 (Leica Biosystems, Illinois) with a 10×/0.3 NA HC PL Fluotar objective (field-of-view of 1.55 mm, working distance of 1 cm, and scan speed of 600 Hz). Glass coverslips with an epoxy ring were used to hold samples for imaging. Samples were placed inside the epoxy ring with a small volume of DBE for refractive index matching. The epoxy ring prevents the DBE from spilling off of the coverslip. One-mm thick cleared brain slices (n=4) were imaged by collecting 52 z-slices, each 10-μm thick, of 1024×1024 images at an excitation of 488 and 633 nm for Tie2-GFP and Lectin-Dylight-649 fluorescence, respectively. Laser power and exposure were set to achieve fluorescence emission intensity histograms of similar shapes across all slices and for both wavelengths. For fluorescence emission collection, the microscope uses a prism and a set of physical sliders to specify the spectral collection band in lieu of a BPF. For Dylight emission imaging, we set the collection band at 695+56  nm. The same Dylight imaging method was used for brain samples from the C57BL/6J.

#### 2.6.2 Whole-brain rendering

An LSM (Z.1, Zeiss) equipped with an s-CMOS PCO.edge camera and two perpendicular objectives for excitation and detection was used to image the whole brain. The sample was submerged in DBE in a custom chamber (Translucence Biosystems, San Diego, California) and suspended from a custom organ holder. This chamber is designed for larger tissue samples and refractive index solutions typically used with tissue clearing methods. The 5×/0.16 (NA) imaging objective (field-of-view of 25 mm, working distance of 18.5 mm) was used with 638- and 488-nm excitation lasers with 640- and 490-nm BP filters for Lectin-Dylight-649 and autofluorescence, respectively. The brain sample is imaged in small regions, or tiles, to obtain high-resolution images of the entire sample. Following manufacturer recommendations, the region captured within each tile overlaps with 20% of the region within adjacent tiles to facilitate subsequent image stitching. Z-stacks (2652×2652  pixel) of 849 slices, each 5.84-μm thick, were acquired for each tile.

### 2.7 Image Processing

#### 2.7.1 Comparison of Tie2-GFP endogenous and Lectin-Dylight-649 exogenous fluorescence

Maximum intensity projections (MIPs) of the two-channel images were made for cross-comparison. Custom-written software in MATLAB (R2020b, The MathWorks, Natick, Massachusetts) was used to randomly select a co-registered subregion of the two-channel MIPs for region of interest (ROI) selection of intravascular and perivascular background regions from the Lectin-Dylight-649 subregion image. Intravascular ROIs were manually selected to a user-defined size. Perivascular background ROIs were also manually selected by users, but with a predefined ROI size of 10×15  pixels. The same ROIs were applied to the co-registered Tie-2-GFP subregion image. For the four ROIs, the mean fluorescence emission intensity was calculated and stored. This process was repeated by three of the authors (D.X., C.C., and B.C.) for a total of 100 times, each time on a new randomly selected subregion of the MIPs.

#### 2.7.2 Whole-brain rendering and three-dimensional visualization

Tiles were manually sorted and stitched with Arivis Tile Sorter (Vision 4D, Arivis AG, Munich, Germany), then saved and converted using the Imaris File Converter (9.3.1, Bitplane, Oxon, UK). Imaris was used to create whole-brain renderings of the cerebrovasculature. Shading was done with the “blend” feature to show surface-level vasculature and “normal shading” to show more internal structures. FIJI was used to create MIPs and for single-slice visualization of vasculature.^22^

### 2.8 Data Analysis

Contrast-to-background ratio (CBR) was calculated as the quotient of the mean fluorescence emission of the vascular ROIs and that of the perivascular background ROIs. All statistical analyses were performed with Prism software (Version 9, GraphPad Software, LLC). A Kruskal–Wallis test with multiple comparisons was used to compare Lectin-Dylight-649 and GFP emission from vascular and background regions. Significance level was set at p=0.05.

## 3 Results

### 3.1 Cerebral Vasculature Visualization with Lectin-Dylight-649

**Fig. 1:**
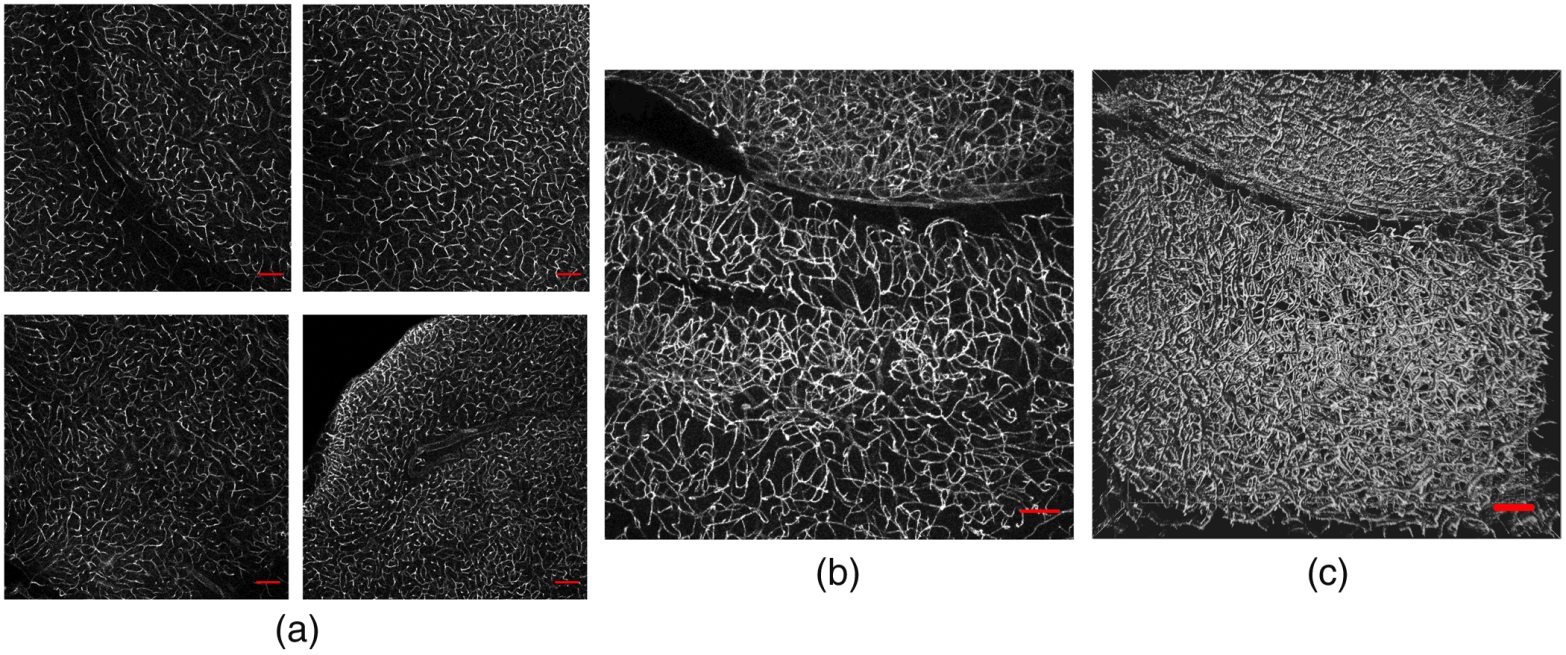
Visualization of Lectin-Dylight-649 in cleared 1-mm-thick coronal brain sections. (a) Single z-stack view of a representative section from different brains (n=4). Scale bar is 100  μm. (b) Representative MIP of 10 fluorescence emission images (60  μm thickness), showing the dense microvascular network. Scale bar is 100  μm. (c) Three-dimensional view of 80 fluorescence emission images taken from a single z-stack. Scale bar is 100  μm. Fly-through rendering of the dataset rendered in three dimensions in (c). A total of 80 fluorescence emission images are contained in this video, spanning a thickness of ∼470  μm. (Video 1, MPG, 1948 KB [URL: https://doi.org/10.1117/1.NPh.8.2.025004.1]).

To validate the efficacy of Lectin-Dylight-649 as a vascular label in cleared tissue, we imaged 1-mm-thick coronal brain sections from C57BL/6J mice. Across different samples from separate animals, the cerebral vasculature is visualized with high contrast [Fig. 1(a)]. Representative MIP [Fig. 1(b)], three-dimensional [Fig. 1(c)], and fly-through views (Video 1) provide complementary visualizations of the dense microvascular network in the mouse brain. Qualitatively, perfusion of Lectin-Dylight-649 provides a robust method for vascular labeling.

### 3.2 Lectin-Dylight-649 Labels Vasculature with High Contrast-to-Background Ratio

**Fig. 2:**
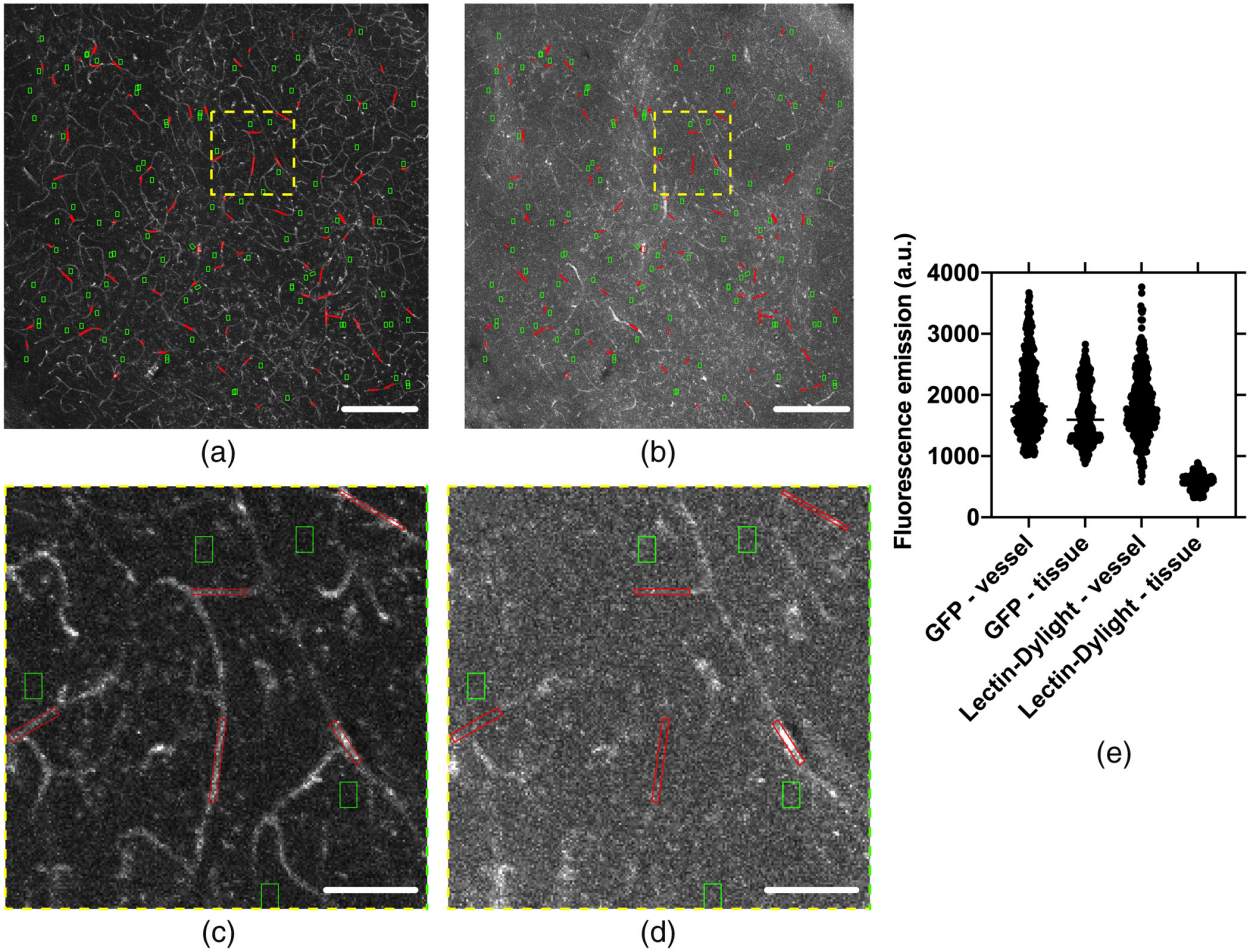
With iDISCO+ optical clearing, Lectin-Dylight-649 labeling exhibits an ∼threefold higher CBR than Tie2-GFP. (a), (b) Lectin and Tie2-GFP fluorescence confocal images, respectively, shown as MIPs taken from the same region of the brain. Both images are on the same colorscale. (c), (d) Representative ROIs taken from (a), (b) in perivascular (green) and vascular (red) regions. (e) Comparison of Lectin and Tie2-GFP intensity profiles in vascular and perivascular regions (n=400 ROIs each).

To evaluate the labeling capacity of perfused Lectin-Dylight-649, we compared its CBR to the Tie2-GFP transgenic vascular model. We measured fluorescence intensities of vascular and background perivascular ROIs for each fluorophore in the same region of dual-labeled brain slices using confocal microscopy (n=4 slices) [Figs. 2(a)–2(d)]. Vascular intensity values were shown to be higher with Lectin-Dylight-649, while background intensity values were higher for Tie2-GFP fluorescence (n=100 ROIs each) [Fig. 2(e)]. Median GFP and Lectin-Dylight-649 fluorescence emission from the vascular regions were similar (p=0.71), but the median GFP and Lectin-Dylight-649 fluorescence emission from the tissue regions differed (mean rank fluorescence emission difference = 820, p<0.0001) [Fig. 2(e)]. The measured fluorescence CBR of Tie2-GFP is ∼three times lower than that of Lectin-Dylight-649 [Fig. 2(e)]. Collectively, these data demonstrate improved vascular visibility with the use of Lectin-Dylight-649 over endogenous GFP, in combination with iDISCO+ optical clearing.

### 3.3 Whole-Brain Cerebral Microvascular Imaging in the Intact Brain

**Fig. 3:**
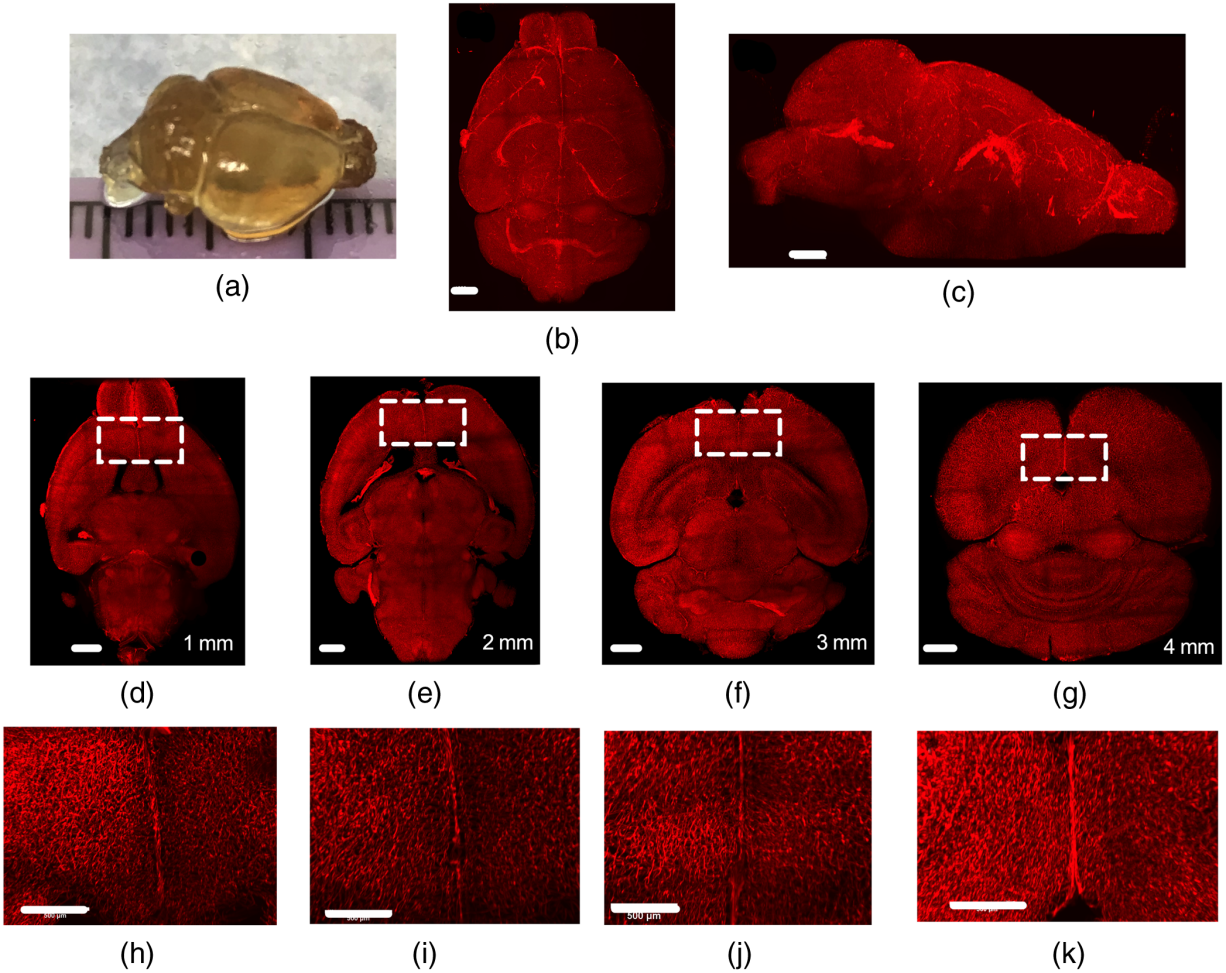
Three-dimensional whole-brain images allow dynamic structure analysis from micrometer to centimeter scales. (a) The entire brain cleared with modified iDISCO+. Renderings of the cerebrovasculature: (b) top view and (c) sagittal view. (d)–(g) 200-μm-thick MIPs of transverse virtual sections taken in steps of 1 mm through the brain. (h)–(k) Magnified view outlined in (d) (scale bar 500  μm). Scale bar in all images=500  μm. Fly-through rendering of 2.2 mm of intact, cleared brain with vasculature labeled with Lectin-Dylight-649. Images collected with the Zeiss Z.1 light-sheet microscope. Imaris software was used to render the dataset (Video 2, MPG, 24,810 KB [URL: https://doi.org/10.1117/1.NPh.8.2.025004.2]). Stitched fly-through rendering of 3.9 mm of an intact, cleared brain labeled with Lectin-Dylight-649. Light-sheet microscope was used with dual-side fusion and pivot scanning to equalize emissions across the whole brain and reduce shadowing effects, respectively. Imaris software was used to stitch and render a 4×3 array of tiles. Total volume size is 31.6  mm×23.6  mm×3.9  mm (Video 3, MPG, 24,132 KB [URL: https://doi.org/10.1117/1.NPh.8.2.025004.3]).

To visualize whole brain microvasculature, Lectin-Dylight-649 was administered via retro-orbital injection followed by cardiac perfusion and the modified iDISCO+ protocol on intact excised mouse brains (n=3). The modified iDISCO+ protocol considerably increased the transparency of the intact brain, compared with its appearance before treatment [Fig. 3(a)]. LSM microscopy yielded several tiles, which were stitched and rendered in three dimensions while maintaining μm resolution over the cm-scale organ. Three-dimensional visualization depicts the detailed cerebrovascular structure [Figs. 3(b), 3(c), Video 2 and Video 3]. Inspection of 200-μm MIPs revealed high-resolution visibility of the vasculature throughout the brain, including internal sections [Figs. 3(d)–3(k)].

## 4 Discussion

Here, we created a simple method to enable visualization of the cerebral microvasculature in an intact mouse brain with micrometer resolution. We found that Lectin-Dylight-649, which is reported to be more stable than the FITC derivatives,^10^^,^^11^ to be an effective method for labeling blood vessels. Our data demonstrate that Lectin-Dylight-649, coupled with a modified iDISCO+ protocol for clearing, achieves superior CBR as compared with endogenous GFP in transgenic Tie2-GFP mice. Preparation of Lectin-Dylight-649 and administration into the bloodstream via retro-orbital injection both are techniques that are simple to master. Thus, our method for vessel labeling should be easy to integrate into studies with other mouse models.

We demonstrated whole-brain imaging and rendering of the cerebral microvasculature is possible with a two-day protocol. We found that using a lectin-based labeling technique and adaptation of the iDISCO+ methodology allowed for a chemically compatible protocol that was achievable in two days. Antibody-based labeling techniques can take ∼one week to complete due to the need to wait for the diffusion of the antibodies and labels from the solution into the tissue sample. The required time can be reduced with the use of tissue sectioning, but with an associated loss of connectivity of features within the individual sections.

Whole-brain vascular visualization was recently demonstrated, as well as the ability to see vasculature in other organs.^14^^,^^15^^,^^17^ To achieve such results, groups used complicated clearing procedures necessitating lengthy labeling techniques (∼weeks) and non-standard equipment (e.g., electrophoretic tissue-clearing chambers and hydrogel embedding equipment), which may impede the routine use of these methods for scientific research.^6^^,^^18^ Our group and others have used cardiac perfusion to administer DiI as a vessel labeling agent.^9^^,^^23^^,^^24^ However, recent work by Salehi et al.^13^ suggests that adjuvant methods (i.e., sodium nitroprusside) to dilate the vasculature is required for DiI to label the vasculature robustly. Our previous data^25^ and data presented here demonstrate that Lectin-Dylight-649 enters the circulatory system via retro-orbital injection and can label vasculature while the animal is alive. We postulate that this *in vivo* vessel labeling approach is a much more straightforward and more effective approach for vessel labeling.

Although the present study describes a translatable protocol for vascular labeling, we did not test the compatibility and visibility of other labels, including fluorophore-conjugated antibodies, with our optical clearing protocol. For example, our previous work centered on FocusClear-based optical clearing has included the use of nuclear stains (i.e., DAPI), fibrillar beta-amyloid (Thioflavin S), and hemosiderin labels (Prussian Blue).^24^^,^^26^ Also, previous work demonstrates that organic solvent-based approaches may quench endogenous fluorescence; thus, the Tie2GFP fluorescence may have been quenched and resulted in lower CBR that we report here.^27^ Future work is required to demonstrate the degree to which fluorescence of Lectin-Dylight-649 is preserved with time.

Future work will involve systematic refinement of our clearing protocol with alternate versions of the “*DISCO” protocol (e.g., FDISCO, SDISCO, and UDISCO). These newer protocols are designed to increase photostability, preserve endogenous fluorescent signals, and clear the whole body.^28^^–^^30^ Additionally, our data suggest that the vessel labeling and clearing approach can be applied to investigate both normal and abnormal vascular architecture in other organs.

## 5 Conclusion

We describe an experimental protocol to visualize the three-dimensional microvascular architecture of the intact mouse brain. The combination of retro-orbital injection of Lectin-Dylight-649 to label the vasculature, the clearing process of a modified iDISCO+ protocol, and light-sheet imaging collectively enables a comprehensive view of the cerebrovasculature. Eliminating additional dehydration/rehydration steps and immersion labeling reduced clearing time to two days. Our protocol is expected to facilitate rapid three-dimensional visualization of the microvascular network for a wide variety of biological and biomedical applications.

## Code, Data, and Materials Availability

Data are available upon reasonable request to the corresponding author.
