# Supplementary material for: Simple methodology to visualize whole-brain microvasculature in three dimensions
Source: Neurophotonics. 2021 Apr 19;8(2):025004. doi: 10.1117/1.NPh.8.2.025004 (PMC8056070; doi:10.1117/1.NPh.8.2.025004)
Supplement: Supplementary file 1 [file NPh_008_025004_SD001.pdf]

SUPPLEMENTARY METHODS AND RESULTS

Comparison of DiI and Lectin-Based Labeling of Cerebral Microvasculature. Two vessel painting agents distinguished by their selective binding sites on cells were compared: (A) DiI-C_{18}, a lipophilic carbocyanine fluorescent dye that embeds itself in the lipid membrane of cells[23]; and (B) *Lycopersicon Esculentum Lectin* (LEL), a glycoprotein with a binding affinity to glycoprotein moieties found in the vascular endothelium[11]. Specifically, we studied LEL bound to one of two fluorescent agents: Fluorescein Isothiocyanate (FITC) or Lectin-Dylight-649. We experimentally compared each of the three agents in the following pairs: (1) DiI-C_{18} with LEL-FITC and (2) DiI-C_{18} with LEL-Dylight-649.

After anesthesia, adult male C3H mice (n=6, 25-30g, Charles River) were administered a retro-orbital injection of an LEL-based agent (FITC-Sigma Aldrich, St. Louis, MO; Dylight649-Vector Labs, Burlingame, CA). Thirty minutes after injection, the mice were euthanized, and cardiac perfusion of saline, DiI-C_{18} (Life Technologies, Grand Island, NY), and 10% formalin was performed. Brains were then harvested and immersed in 10% formalin for 24 hours and transferred to a PBS with 0.02% sodium azide solution for fluorophore preservation. Coronal sections of 0.5mm thickness were then prepared using a brain matrix slicer.

Brain samples were optically cleared by complete immersion of sections in FocusClear (CelExplorer Labs, Hsinchu, Taiwan) for a minimum of 6 hours. We used FocusClear due to 1) an incompatibility between DiI-C_{18} and chemical agents used with the iDISCO protocol, resulting in the extraction of DiI-C_{18} from the vasculature; and 2) our previous experience with it as a brain clearing agent[24-26,31].

SUPPLEMENTARY FIGURES

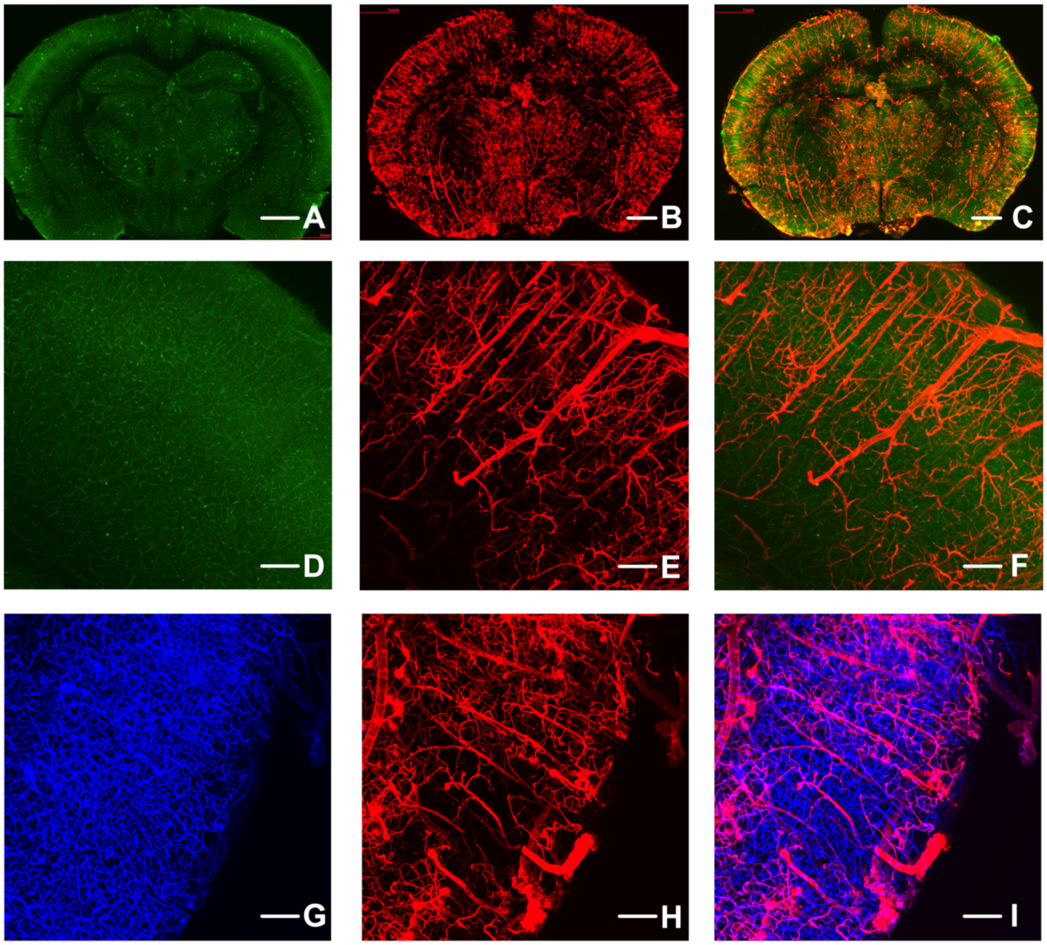

Figure S1 Representative maximum intensity projection images of coronal brain sections. (A-C) (A) Fluorescence emission image of a section labeled with lectin-FITC. (B) Fluorescence emission image of DiI-C_{18} in the same brain section as in (A). (C) Co-registered fluorescence emission image of both lectin-FITC (green) and DiI-C_{18} (orange-red). (D-F) Magnified fluorescence emission images of the same images in (A-C). (D) shows extensive lectin-FITC labeling of the microvasculature but considerable background fluorescence emission, resulting in relatively poor vascular contrast. (E) shows good vascular contrast in larger microvessels but poor labeling of small microvessels. (F) shows the strengths and limitations of lectin-FITC and DiI-C_{18} as exogenous vascular labels. (G-I) Magnified fluorescence emission images of a representative brain section from a different mouse, with vessels labeled with both lectin-Dylight-649 and DiI-C_{18}. (G) shows extensive lectin-Dylight-649 labeling of the microvasculature and good vascular contrast of microvessels. Similar to (E), (H) shows good vascular contrast in larger microvessels but poor labeling of small microvessels. (I) highlights the overall improvement in vessel labeling with lectin-Dylight-649. Scale bars: (A-C) 1 mm, (D-I) 100 µm.

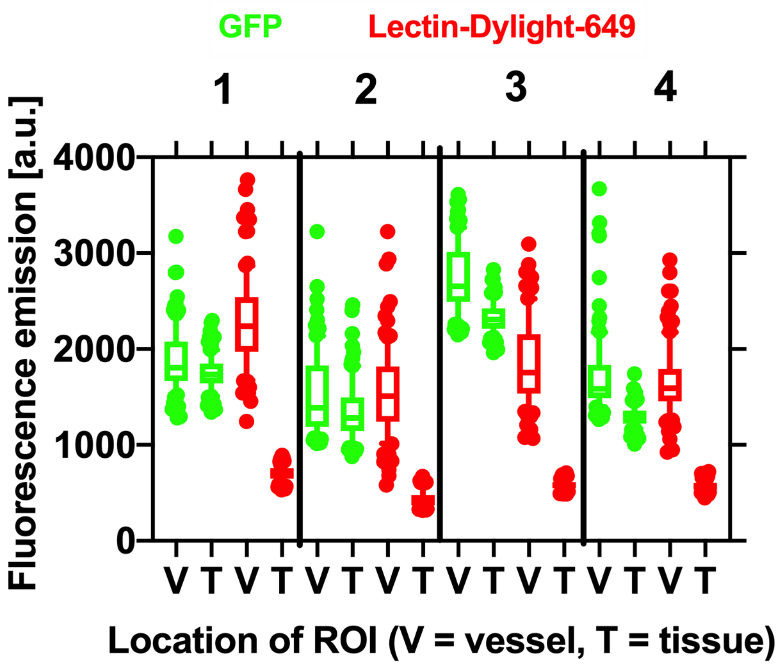

Figure S2 Fluorescence emission of GFP (green) and lectin-Dylight-649 (red) from each coronal brain slice (n=4). Compiled data are shown in Figure 2(E). Each column of data represents the summary of 100 ROIs, as individual box-and-whisker plots. ROIs were selected in vessel regions (V) and in perivascular tissue (T). For each slice, the GFP and lectin-Dylight-649 ROIs are co-registered. The median value is shown as a horizontal line in the box, and the edges of the boxes represent the 10th and 90th percentile fluorescence emission values. The filled circles represent the remaining fluorescence emission values. For each slice, the median vessel fluorescence emission was higher than the tissue emission for both GFP and lectin-Dylight-649 (Mann-Whitney test, $p < 0.01$), but the vessel contrast-to-background ratio was considerably higher for lectin-Dylight-649 (range 2.8-3.7) than for GFP (range 1.0-1.2).
